# On the Indian Thrush

**Published:** 1841-10

**Authors:** 


Art. VI.—*Over de Indische Sprouw* (*aphthæ orientales.*) Door W. Bosch, Chirurgijn-Majoor bij het leger in de Oost-Indiën, &c.—*Amsterdam*, 1837.

*On the Indian Thrush.* By W. Bosch, Surgeon-Major in the Army in the East Indies.—*Amsterdam*, 1837. 8vo, pp. 60.

This little work is addressed chiefly to young medical practitioners in the Dutch possessions in India, to acquaint them with the nature of a dangerous and peculiar disease there prevalent. The author divides his history of it into three periods, each of which however is, in different cases, of different duration. In the first stage there are loss of appetite, weight at the stomach, and nausea, and the tongue is clean or covered with a yellowish fur on the right side. The expression of the countenance is natural, the eyes bright and light yellow, the perspiration diminished, the pulse pretty natural, the intestinal evacuations irregular and bilious, and mixed with undigested substances. The abdomen is distended, the epigastrium tender, the urine red and scanty. In the second stage, which is regarded as inflammatory, all the preceding symptoms are increased; there is more fever, a quantity of gas and acid in the stomach, and evident torpor of the liver. In this stage also there is observed a great tenderness with redness of the tongue, mouth, and fauces. All these disorders are in the third stage still more increased, so that nothing can be taken into the mouth but mild mucilaginous substances, and there now come on excessive and colliquative diarrhœa of citron-yellow or gray fluid, with local, fetid, and rapidly exhausting sweats, and with scanty and very acrid urine.

The author regards the ganglionic nervous system as the essential seat of the disease; and ascribes, as its predisposing causes, partly the climate of Padang, which is situated on the west coast of Sumatra, and is frequently damp and has a very variable temperature, and partly the food, which in great part consists of raw potatoes. The proximate cause of the disease is considered to be a cachectic condition of the fluids and a relaxed state of the solids of the body.

In reference to treatment, the author states that he disapproves of emetics, but can recommend gentle purgatives to be administered on first seeing the patient. Calomel is not tolerated, but mercurial ointment may be rubbed in over the upper part of the abdomen with great advantage. At a later period diluted sulphuric acid is useful, but only in very small doses and given with great circumspection. At the close of the disorder, tonics, especially the lichen islandicus, are advantageous. Of narcotics, the extract of hyoscyamus and the aqua laurocerasi are beneficial. Among external remedies, the best are leeches applied in small numbers; and for counter-irritation, tartar emetic dissolved in water and applied so as to produce an eruption. Galvanism produced no effect. The treatment of the disease however is, like that of all diseases in which there is increased action with diminished power, difficult. The transition to the adynamic state takes place easily, and either stimulants or antiphlogistics must be administered with the greatest reserve; the first soon bring on exhaustion by their indirect action, the last by their direct influence.

